# Mathematical Modeling of Cancer Invasion: The Role of Membrane-Bound Matrix Metalloproteinases

**DOI:** 10.3389/fonc.2013.00070

**Published:** 2013-04-03

**Authors:** Niall E. Deakin, Mark A. J. Chaplain

**Affiliations:** ^1^Division of Mathematics, University of DundeeDundee, UK

**Keywords:** cancer invasion, mathematical model, metalloproteinases, membrane-bound MMPs, extracellular matrix

## Abstract

One of the hallmarks of cancer growth and metastatic spread is the process of local invasion of the surrounding tissue. Cancer cells achieve protease-dependent invasion by the secretion of enzymes involved in proteolysis. These overly expressed proteolytic enzymes then proceed to degrade the host tissue allowing the cancer cells to disseminate throughout the microenvironment by active migration and interaction with components of the extracellular matrix (ECM) such as collagen. In this paper we develop a mathematical model of cancer invasion which consider the role of matrix metalloproteinases (MMPs). Specifically our model will focus on two distinct types of MMP, i.e., soluble, diffusible MMPs (e.g., MMP-2) and membrane-bound MMPs (e.g., MT1-MMP), and the roles each of these plays in cancer invasion. The implications of MMP-2 activation by MMP-14 and the tissue inhibitor of metalloproteinases-2 are considered alongside the effect the architecture of the matrix may have when applied to a model of cancer invasion. Elements of the ECM architecture investigated include pore size of the matrix, since in some highly dense collagen structures such as breast tissue, the cancer cells are unable to physically fit through a porous region, and the crosslinking of collagen fibers. In this scenario, cancer cells rely on membrane-bound MMPs to forge a path through which degradation by other MMPs and movement of cancer cells becomes possible.

## Introduction

For metastasis to occur, cancer cells must exhibit invasion through a variety of structured media such as the highly dense collagen constitution of some peritumoral stroma (Hanahan and Weinberg, 2000, 2011). This can occur by the secretion of enzymes that are capable of degrading components of the ECM or by the adoption of an amoeboid phenotype that allows cancer cells to travel through the medium in a protease-independent manner (Friedl and Wolf, 2003; Sahai, 2005).

Mathematical modeling of solid-tumor growth dates back to the works of Thomlinson and Gray (1955) and Burton (1966), where oxygen was modeled as a nutrient diffusing from the outer edge (boundary) of a tumor inwards to investigate its role in necrosis. Mathematical modeling has continued to develop to investigate a number of topics in cancer progression and invasion, including models taking into account: oxygen/nutrient driven dynamics, the immune response, the acidity of the environment, force-based pressure, the microenvironment in general, and protease-dependent invasion using the techniques of partial differential equations for densities of cells, individual-based models including cellular automaton models and multi-scale models as outlined in the review papers of Araujo and McElwain (2004), Rejniak and McCawley (2010), and Lowengrub et al. (2010) and the references therein.

In this paper, we focus on a continuum, deterministic approach to protease-dependent invasion where matrix degrading enzymes cleave collagen fibrils and other ECM components rather than protease-independent invasion where mechanical forces physically displace matrix fibrils and cancer cells adopt an amoeboid-like shape (epithelial-mesenchymal transition). The microenvironment of the tumor plays a significant role in cancer progression with matrix metalloproteinases acting as regulators, allowing obstacles to be overcome (Rowe and Weiss, 2009; Kessenbrock et al., 2010).

Matrix metalloproteinases (MMPs) are one mechanism by which cancer cells have the ability to invade. These proteolytic enzymes are a family of 23 enzymes in humans which have the capacity to degrade virtually all components of the surrounding tissue (Kleiner and Stetler-Stevenson, 1999). This in turn facilitates cancer growth and spread by virtue of the available space left in the absence of the degraded ECM as well as by other proteins that are released by the degraded tissue encouraging the cancer growth (Werb, 1997; López-Otín and Overall, 2002). Haptotaxis is a process of directed cell migration whereby cells move in response to gradients of adhesion sites naturally present in the ECM.

MMPs are zinc-dependent endopeptidases whose main function is in the regular turnover of the ECM (Nagase and Woessner, 1999). This process is exploited in cancer growth and invasion where various MMPs are over expressed. The expression of MMPs faces control at the level of transcription but can also face inhibition when moving from the proMMP state to an active MMP as well as inhibition when it exists in its active state.

The family of MMPs can exhibit both pro- and anti-invasive characteristics (Nöel et al., 2012) but in this paper we focus on the pro-invasive MMPs of MMP-2, MMP-9, and MT1-MMP. MMP-2 and MMP-9 are secreted in their inactive zymogen forms of proMMP-2 and proMMP-9 whereas the fully active MT1-MMP is expressed on the cell surface after being activated internally. The activation process by which the various MMPs develop into their fully active form differs between them. This interplay between the enzymes is emphasized by the coexpression of proMMP-2, MT1-MMP, MMP-2, and TIMP2 in a variety of tissues (Kinoh et al., 1996). While MT1-MMP was initially thought to have activity limited to activating MMP-2, it has since been found to also have a direct role in tissue degradation (d’Ortho et al., 1997).

The main constituent of the stroma is the structural, cross-linked type I collagen. MT1-MMP exhibits strong type I collagenolytic capabilities and weak gelatinolytic capabilities. Conversely, MMP-2 exhibits weak type I collagenolytic capabilities and strong gelatinolytic capabilities (Tam et al., 2004) where it is unable to degrade cross-linked collagen type I and type IV but is able to degrade the uncross-linked variants (Zhang et al., 2013). MMP-2 can, however, critically degrade type IV collagen, the main component of the basement membrane and an extracellular barrier. As MT1-MMP is bound to cancer cells, its region of proteolytic activity is more restricted than that of the freely diffusive proteolytic enzyme MMP-2. This means that tissue degradation in advance of the cancer cells is the result of the soluble MMPs. While MT1-MMP activity is restricted in range, it has an advantage in its capability of overcoming environments of higher collagen density such as exists in some peritumoral stroma. Sabeh et al. (2009) have shown that when cancer cells are faced with structural barriers created in reconstituted gels by covalently cross-linked fibrils of type I collagen, or that exist in the stromal environment of the mammary gland, invasion is dependent on MT1-MMP-mediated proteolysis.

Other mathematical models that have examined the activation of MMP-2 by MT1-MMP include Karagiannis and Popel (2004), Donzé et al. (2011), and Hoshino et al. (2012). Karagiannis and Popel (2004) developed a set of non-spatial ordinary differential equations of this activation system and examined the interplay between the activation system of MMP-2 and ectodomain shedding of MT1-MMP. They found that in the absence of TIMP2, ectodomain shedding of MT1-MMP dominated dynamics but that introducing TIMP2 would encourage MMP-2 activation and protect MT1-MMP from shedding. They later developed their model to examine proteolysis during the migration of a tip endothelial cell as is relevant in angiogenesis (Karagiannis and Popel, 2006). Donzé et al. (2011) performed global robustness and sensitivity of the model and explored the possibility of oscillatory dynamics in the system. Hoshino et al. (2012) developed their extensive model of all possible interactions of enzymes using A-Cell (Ichikawa, 2001). They provide experimental validation alongside their computational model used to investigate the significance of turnover of MT1-MMP for proteolysis at invadopodia. In this paper, we apply a reduced form of the activation system of MMP-2 to our model of cancer cell invasion where tissue degradation is mediated by either MT1-MMP or MMP-2.

The architecture of the ECM plays a pivotal role when considering cancer cell invasion. Li et al. (2008) and Sabeh et al. (2009) have made attempts to create *in vitro* environments that better represent those found *in vivo* by embedding multicellular spheroids of HT-1080 fibrosarcoma cells within gels of cross-linked native type I collagen. Both studies found that MT1-MMP silencing blocks virtually all collagenolytic and invasive activity. In this paper, we provide an approach that considers what effect the architecture of the ECM, such as pore size of tissue and proportion of ECM made up of cross-linked collagen, may have when applied to a model of cancer invasion. We are able to consider a heterogeneous ECM and incorporate haptotaxis as occurring only in response to ECM gradients created by the release of enzymes such as MMPs.

In this paper therefore, we develop a mathematical model of cancer invasion which consider the role of matrix metalloproteinases (MMPs). Specifically our model will focus on two distinct types of MMP, i.e., soluble, diffusible MMPs (e.g., MMP-2), and membrane-bound MMPs (e.g., MT1-MMP), and the roles each of these plays in cancer invasion. Our model will also consider the influence of the structure of the matrix on cancer cell invasion and to achieve this (using a continuum PDE model) we will introduce the concept of a “matrix suitability modifier.”

## Materials and Methods

**Figure 1:**
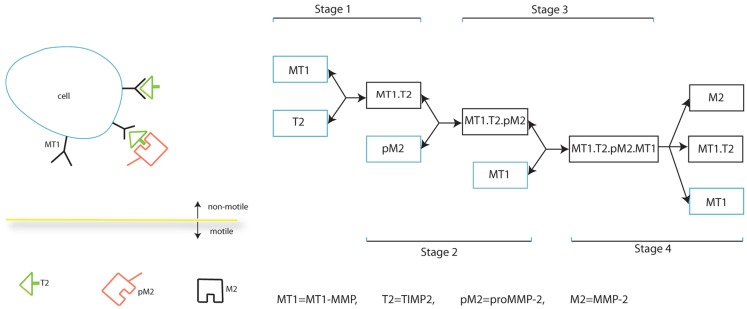
**Schematic diagram of MMP-2 activation**.

In this section, we present our mathematical model which describes the interplay between MMPs in cancer invasion, specifically MT1-MMP activation of MMP-2, the balance between TIMP2 inhibition of both MT1-MMP and MMP-2, and the dual role of TIMP2 as inhibitor of species and activator of MMP-2. The full process of MMP-2 activation is shown in Figure 1. The species/complexes in a blue box are produced while those in the black box are simply formed. Whether a species/complex is free to move, without considering lateral diffusion on a cell and the relative movement of a cell, is also indicated.

**Figure 2:**
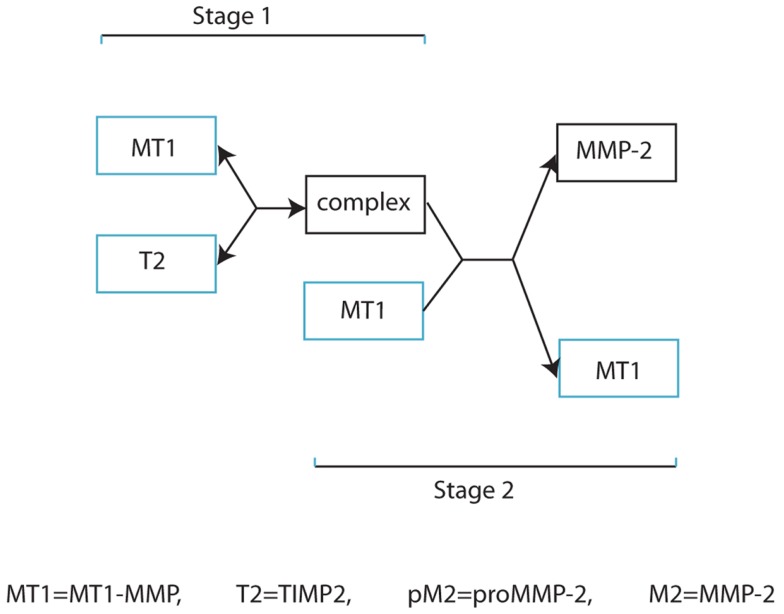
**Simplified schematic diagram of MMP-2 activation**.

Our invasion model is based on a simplified form of MMP-2 activation as outlined in Figure 2, and involves considering stages 2, 3, and 4 of Figure 1 as a single process. This retains the key details of whether a complex is stationary or not in relation to cell movement and is a significant factor considering the relative speed of binding of the freely diffusive TIMP2 to a complex bound to the cell. However, we feel that this simplification of the process is appropriate in capturing the dynamics of the two functional forms by which invasion is facilitated. A basic model is presented in the Supplementary Material to clarify the difference between these two functional forms of invasion mediated by the highly localized tissue degradation by MT1-MMP and the more extensive tissue degradation by the diffusible MMP-2.

In our model we denote by *c*(**x**, *t*) the cancer cell density, *v*(**x**, *t*) the ECM density, *m_s_*(**x**, *t*) the MMP-2 concentration, and by *m_t_*(**x**, *t*) the MT1-MMP concentration. In addition, we let *T*(**x**, *t*) denote the TIMP2 concentration, *f*(**x**, *t*) the concentration of the complex of MT1-MMP:TIMP2 (with an assumed proMMP-2 attached) and *s*(**x**, *t*) the “matrix suitability modifier.” The matrix suitability modifier acts as an environmental factor by reducing the level of a cell population that can physically move through the matrix – as an extension of the volume-filling principles – by taking into account the pore size of the collagen substrate along with reducing the amount of matrix that is considered available to be degraded. In this model, we consider a matrix environment with a neutral effect on these processes to have a matrix suitability modifier value of 1, which results in the standard dynamics expected from such a model. A matrix environment containing difficult regions of ECM for cancer cells to invade will be represented by a matrix suitability modifier with a value 0 ≤ *s* < 1, with values toward zero describing an environment that is more difficult for cancer cells to navigate through, as well as a reduction in ECM that is available to be degraded. We also impose the condition that *s* + *v* ≥ 1, otherwise instead of limiting movement and tissue degradation, movement would be encouraged in the opposite direction and the ECM degradation term would cause the density of ECM to increase.

As both MT1-MMP and the intermediate complex are bound to cancer cells, they experience movement in the same direction as the cancer cells and with a magnitude determined by the concentration of enzymes at that location. We consider both MMP-2 and MT1-MMP to undergo natural decay. We include collagen-induced MT1-MMP expression in our model through a function α*_mt_c*(1 + *v*) (Zigrino et al., 2001). This may take into account observations showing that collagen-dense mouse mammary tissues result in cancer cells with a more invasive phenotype Provenzano et al. (2008). We also include volume-filling of the form (1 − *c* − *v*) to represent the competition for space between the cancer cells and ECM. This is applied both to the haptotactic response of cancer cells to ECM gradients and to the growth rates for both the cancer cell and ECM populations. Finally, we have an ECM remodeling rate of μ*_v_*(1 − *c* − *v*) (cf. Gerisch and Chaplain (2008)). The full model is therefore given as:

(1) $$\begin{array}{llll} \frac{\partial c}{\partial t}= & \;\nabla \left(D_{c}\nabla c-\chi c\left(s-1+v\right)\left(1-c-v\right)\nabla v\right) & & \\ & +\mu_{c}c\left(1-c-v\right), & \end{array}$$

(2) $$\begin{array}{lll} \frac{\partial v}{\partial t}= & \;-\delta \left(s-1+v\right)\left(m_{s}+m_{t}\right)+\mu_{v}\left(1-c-v\right), & \end{array}$$

(3) $$\begin{array}{lll} \frac{\partial m_{s}}{\partial t}= & \;\nabla \left(D_{m_{s}}\nabla m_{s}\right)-\phi_{31}Tm_{s}+\phi_{32}m_{t}f-\beta_{m_{s}}m_{s}, & \end{array}$$

(4) $$\begin{array}{llll} \frac{\partial m_{t}}{\partial t}= & \;m_{t}\nabla \left(D_{c}\nabla c-\chi c\left(s-1+v\right)\left(1-c-v\right)\nabla v\right)-\phi_{41}Tm_{t} & & \\ & +\phi_{42}-\beta_{m_{t}}m_{t}+\alpha_{m_{t}}c\left(1+v\right), & \end{array}$$

(5) $$\begin{array}{lll} \frac{\partial T}{\partial t}= & \;\nabla \left(D_{T}\nabla T\right)-\phi_{51}Tm_{s}-\phi_{52}Tm_{t}+\phi_{53}f+\alpha_{T}c, & \end{array}$$

(6) $$\begin{array}{llll} \frac{\partial f}{\partial t}= & \;f\nabla \left(D_{c}\nabla c-\chi c\left(s-1+v\right)\left(1-c-v\right)\nabla v\right)+\phi_{61}Tm_{t} & & \\ & -\phi_{62}fm_{t}-\phi_{63}f, & \end{array}$$

(7) $$\begin{array}{lll} \frac{\partial s}{\partial t}= & \;\delta_{s}m_{t}\left(1-s\right). & \end{array}$$

**Figure 3:**
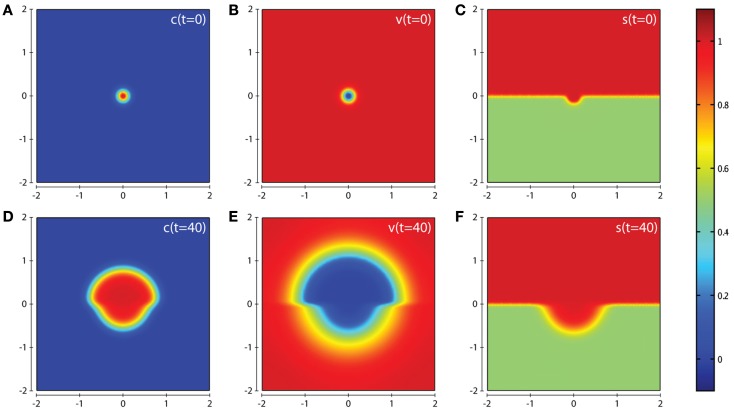
**Plots showing the simulation results obtained in a two-dimensional domain where asymmetric invasion of the ECM is achieved by the cancer cells**. We use the matrix suitability modifier *s* to represent a medium with neutral abilities in the upper half of the region (*s* = 1; red) and with a reduced, moderate, suitability for invasion in the lower half (*s* = 0.5; green). Plots **(A–C)** show the initial values of the cancer cell and ECM densities as well as the initial layout of the matrix suitability modifier with **(D–F)** showing their resultant profiles at *t* = 40 (corresponding to ∼4.6 days). Simulations are performed using the baseline parameter set.

In all computational simulations, we apply zero-flux boundary conditions to equations (1), (3–6). The initial conditions imposed depend on the precise invasion scenario we are considering. In our the first invasion scenario (cf. Figure 3) we have a cluster of cancer cells in the center of a homogeneous ECM with a small amount of activated enzymes already released, i.e., $c\left(0\right)=e^{\left(\frac{-\left(x^{2}+y^{2}\right)}{0.02}\right)}$, *v*(0)=1 − *c*(0)v(0)=1-c(0), *m_s_*(0) = *m_s_*(0) = *T*(0) = *f*(0) = *c*(0)$m_{s}\left(0\right)=m_{t}\left(0\right)=T\left(0\right)=f\left(0\right)=c\left(0\right)$. The initial condition for the matrix suitability modifier *s* is best seen in Figure 3C. The initial conditions used for *c*, *v*, *s* in the second invasion scenario are best seen from the plots in Figures 7A–C, and also in this case *m_s_*(0) = *m_t_*(0) = *T*(0) = *f*(0) = *c*(0).

### Parameter estimation

We non-dimensionalize the model using the reference variables *τ* = 10^4^ s and *L* = 0.1 cm. While the reference enzyme concentration can be difficult to obtain, we take it be 1 nM with concentrations throughout the considered timeframe to be within the range 0–25 nM. Tutton et al. (2003) find pre-operative MMP-2 levels in plasma of colorectal cancer patients of 568.9 ng/ml = 7.89 nM while Butler et al. (1998) and English et al. (2001) performed experiments with enzyme concentrations of order 100 nM. The parameters β*_ms_*, β*_mt_*, α*_mt_*, and α*_T_* were therefore chosen so that the concentrations of active MT1-MMP and MMP-2 are in the range 0–25 nM. The baseline parameter set presented in Table 1 is used for all computational simulations of the model unless otherwise specified.

**Table 1:** **Baseline parameter set for the model**.

	Non-dimensional form	Original	Unit	Reference
*D_c_*	3.5 × 10^-4^			Anderson et al. (2000)
χ	5 × 10^-3^			Anderson et al. (2000)
μ*_c_*	0.3			Anderson et al. (2000)
δ	1			Anderson et al. (2000)
μ*_v_*	0.2			Anderson et al. (2000)
*D_ms_*	1.29 × 10^2^	1.29 × 10^8^	cm^2^s^−1^	Collier et al. (2011)
*Φ*_31_	5	5 × 10^5^	M^−1^s^−1^	Estimated
*Φ*_32_	0.195	1.95 × 10^4^	M^−1^s^−1^	Estimated
β*_ms_*	0.1 D	1 × 10^-5^	s^−1^	Estimated
α*_mt_*	5	5 × 10^-4^	s^−1^	Estimated
*Φ*_41_	27.4	2.74 × 10^6^	M^−1^s^−1^	Toth et al. (2000)
*Φ*_42_	2	2 × 10^-4^	s^−1^	Toth et al. (2000)
β*_mt_*	0.1	1 × 10^-5^	s^−1^	Estimated
*D_T_*	1.29 × 10^2^	1.29 × 10^8^	cm^2^s^−1^	Collier et al. (2011)
α*_T_*	4	4 × 10^-4^	s^−1^	Estimated
*Φ*_51_	5	5 × 10^5^	M^−1^s^−1^	Estimated
*Φ*_52_	27.4	2.74 × 10^6^	M^−1^s^−1^	Toth et al. (2000)
*Φ*_53_	2	2 × 10^-4^	s^−1^	Toth et al. (2000)
*Φ*_61_	27.4	2.74 × 10^6^	M^−1^s^−1^	Toth et al. (2000)
*Φ*_62_	0.195	1.95 × 10^4^	M^−1^s^−1^	Estimated
*Φ*_63_	2	2 × 10^-4^	s^−1^	Toth et al. (2000)
δ*_s_*	0.025			Estimated

## Results

In this section we present the computational simulation results of our invasion model equations (1–7) in a 2-dimensional domain (all parameter values are from the baseline set). The first scenario we consider is one in which the tissue is considered to have neutral effects on invasion in the top half of the domain by having a matrix suitability modifier *s* = 1, with the lower half of the domain having moderate characteristics limiting invasion by having the matrix suitability modifier of $s=\frac{1}{2}$, as shown in Figure 3C. The value of $s=\frac{1}{2}$ may represent a region that contains a tissue where (i) half the constituent parts of the ECM are cross-linked collagen, (ii) half the considered ECM has a pore size below a threshold α that blocks invasion, (iii) more than half the considered ECM has a pore size in the range α–β that slows invasion, or (iv) some combination of the factors presented in (i–iii) that has the equivalent effect. As can be seen from the plots in Figure 3 we observe an asymmetric invasion by the cancer cells, with a reduced invasion in the lower half of the domain (cf. Figure 3D) and also a reduced degradation of ECM in the lower half of the domain (cf. Figure 3E).

**Figure 4:**
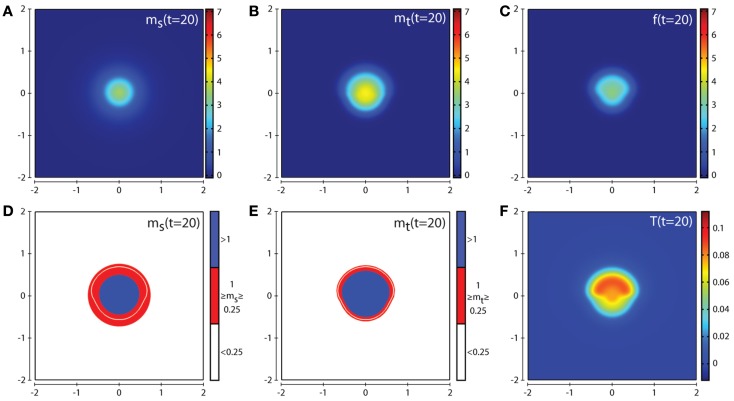
**Plots showing the simulation results obtained in a two-dimensional domain where asymmetric invasion of the ECM is achieved by the cancer cells**. The concentrations of MMP-2, MT1-MMP, the intermediary complex *f*, and TIMP2 at *t* = 20 (corresponding to ∼2.3 days) are shown in plots **(A–C,F)** respectively. Plots **(D,E)** show the MMP-2 and MT1-MMP concentrations at *t* = 20 with appropriate thresholds near the invasive front of the cancer cell invasion. The white contour line shows the cancer cell density at level 0.01 chosen to represent the maximum extent of invasion. Simulations are performed using the baseline parameter set.

**Figure 5:**
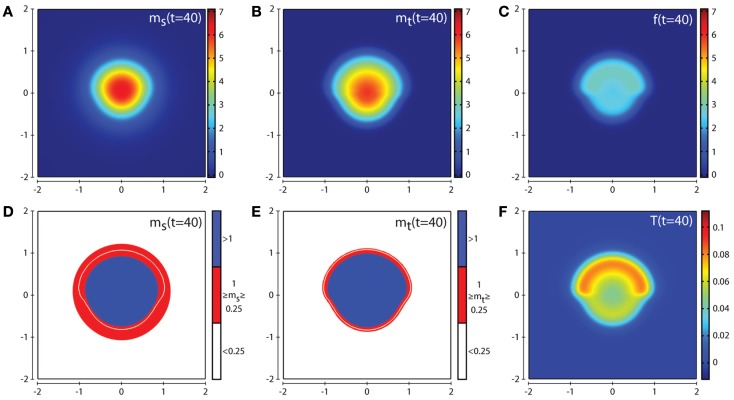
**Plots showing the simulation results obtained in a two-dimensional domain where asymmetric invasion of the ECM is achieved by the cancer cells**. The concentrations of MMP-2, MT1-MMP, the intermediary complex *f*, and TIMP2 at *t* = 40 (corresponding to ∼4.6 days) are shown in plots **(A–C,F)** respectively. Plots **(D,E)** show the MMP-2 and MT1-MMP concentrations at *t* = 40 with appropriate thresholds near the invasive front of the cancer cell invasion. The white contour line shows the cancer cell density at level 0.01 chosen to represent the maximum extent of invasion. Simulations are performed using the baseline parameter set.

Figures 4 and 5 show the corresponding evolution of the various enzyme concentrations using the baseline parameter set. The plots in Figures 4F and 5F show that any free TIMP2 that is produced or released from a complex is quickly bound to either free MT1-MMP or MMP-2. The plots in Figures 4D and 5D show that while MMP-2 can freely diffuse throughout the environment, its profile is affected by the source term coming from the asymmetric cancer cell invasion dynamics. The plots in Figures 4E and 5E show how the degradative effect of MT1-MMP is limited by its dependence on transport by the cancer cells. This is demonstrated by a reduced invasive profile in the bottom half of each plot.

**Figure 6:**
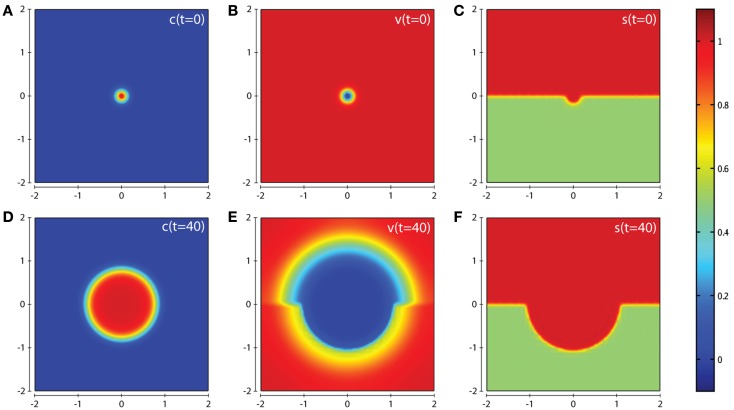
**Plots showing the simulation results obtained in a two-dimensional domain where the suitability modifier *s* represents a medium with neutral abilities in the upper half of the region (*s* = 1; red) and a reduced, moderate, suitability for invasion in the lower half (*s* = 0.5; green)**. We consider a scenario where the suitability modifier is rapidly brought back to a neutral state by performing simulations using the baseline parameter set with the exception of δ*_s_* = 10. Plots **(A–C)** show the initial values of the cancer cell and ECM densities as well as the initial layout of the matrix suitability modifier with plots **(D–F)** showing their resultant profiles at *t* = 40 (corresponding to ∼4.6 days). As can be seen, in this case there is an almost symmetric invasion pattern. This shows the influence of the parameter δ*_s_* on invasion.

In Figure 6 we examine an invasive scenario where the effects of the suitability of the matrix play little or no role. This is achieved by increasing the parameter δ*_s_* to a value of 10 (all other parameters kept at baseline values and using the same initial conditions as in Figures 3 and 4). This scenario represents a region of tissue that is difficult to invade and degrade by MMP-2 alone in the lower half of the domain, but one that is rapidly remodeled when in range of MT1-MMP to a condition that is considered to have a neutral effect on invasion. Under these conditions, it can be seen from the plot in Figure 6D that the cancer cells invade in a symmetric manner (unlike the scenario in Figure 3D). However, we can also see from the plot in Figure 6E that there is a reduced degradation of ECM due to MMP-2 in the lower half of the domain compared with the upper half. This shows the significance of the rate that MT1-MMP is able to remodel the ECM in the spatial layout of the tumor. The corresponding plots of the concentrations MMP-2, MT1-MMP, the intermediary complex *f*, and TIMP2 at *t* = 20 and *t* = 40 are given in Figures S2 and S3 of the Supplementary Material.

**Figure 7:**
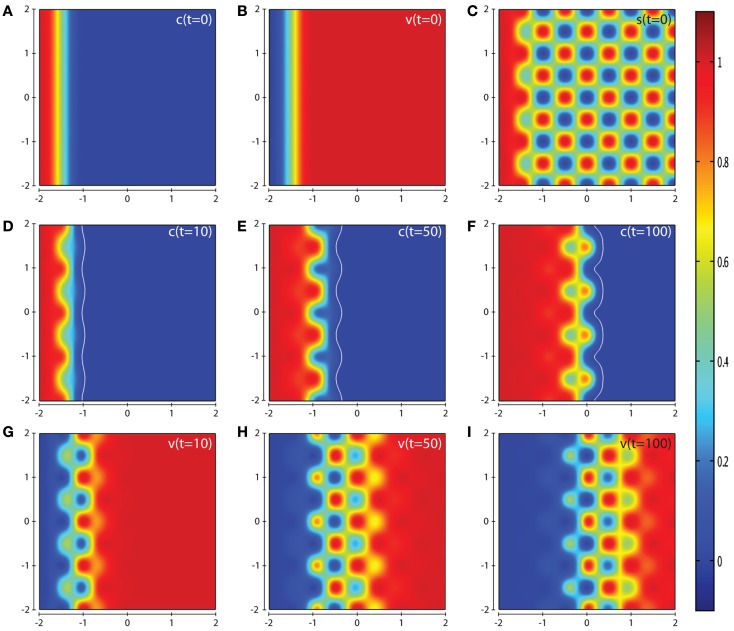
**Plots showing the simulation results obtained in a two-dimensional domain with a spatially complex matrix suitability modifier *s* to more accurately depict the observations of certain *in vivo* experiments**. Plots **(A–C)** show the initial values of the cancer cell and ECM densities as well as the initial structure of the matrix suitability modifier. Plots **(D–F)** show the resultant profiles of cancer cell density at *t* = 10, 50, and 100 (corresponding to ∼1.15, 5.75, and 11.5 days, respectively). The white contour line shows the cancer cell density at level 0.01 chosen to represent the maximum extent of invasion. Plots **(G–I)** show the resultant profiles of ECM density at *t* = 10, 50, and 100. The simulations were performed using the baseline parameter set with the exception of the parameter δ*_s_* = 0.0025. The plots show a highly heterogeneous invasion pattern at the invasive front, once again highlighting the role of the matrix suitability modifier *s* and the parameter δ*_s_*.

In Figure 7, we present the computational simulation results of cancer cell invasion in a more heterogeneous environment such as would be expected in certain *in vitro* experiments (and also *in vivo*). For this scenario, we used the baseline parameter set, except for the parameter δ*_s_* which is reduced by a factor of ten to a value of 0.0025. The plots in Figures 7D–F show that the cancer cells take a longer time to invade the less suitable regions of ECM resulting in a heterogeneous invasion pattern. In Figure 7F, we can see that there are regions of higher cancer cell density (small red zones) in advance of regions of lower cancer cell density (small green zones) but without having broken off from the main mass entirely. The corresponding plots of the concentrations of MMP-2 and MT1-MMP at *t* = 10, 50, 100 are given in Figure S4 of the Supplementary Material. The effect of varying the parameter δ*_s_* can be seen by comparing the results in Figure 7 with Figure S5 in the Supplementary Material, where we have used the default parameter set but we do not observe any regions of high cancer cell density in advance of regions of lower cancer cell density.

## Discussion

In this paper we have presented a mathematical model for cancer invasion of tissue, focusing on the roles of soluble and bound MMPs. Additionally we introduced the concept of “matrix suitability,” governed by the variable *s* in our model. By considering the suitability of the matrix as a factor affecting ECM degradation and the movement of enzymes and cancer cells, we were able to generate heterogeneity in the ECM caused solely by matrix degradation. This meant we were able to focus on the effects of these gradients explicitly caused by matrix degradation rather than ECM density gradients due to intrinsic tissue heterogeneity. The computational simulation results showed that the matrix suitability modifier and its regulation played an important role in determining the precise pattern of invasion. As has been observed in the experimental data of Sabeh et al. (2009) and Li et al. (2008), we have shown that the architecture of the tissue can negatively impact invasion under circumstances of pore size being below an optimal level or in environments of cross-linked collagen type I and IV, with both of these conditions requiring tissue remodeling specifically by MT1-MMP. In addition to this, invasion is reduced where TIMP2 is overly produced. To investigate the matrix suitability modifier from a biological perspective, experiments would need to be carried out to obtain the initial layout of the suitability modifier as well as the parameter δ*_s_*. The first step in doing this would be to find out the effects of different tissue pore size on cancer cell migration to establish what range of pore sizes would be considered a neutral modifier, what range of pore sizes allow migration at reduced levels, and what range of pore sizes completely block migration. This could be done by using the approaches of Nyström et al. (2005) and Martins et al. (2009) where they performed *in vitro* experiments using a collagen:matrigel assay to investigate the invasiveness of cancer cells to establish a quantitative “invasive index” in organotypic cultures. Once there is quantitative data for these effects, obtaining data on the structure of the tissue through effective imaging techniques such as those described in Wolf et al. (2009) would allow one to generate realistic initial conditions of the matrix suitability modifier. An estimate of the parameter δ*_s_* could then be obtained by validating the model against experiments similar to those found in Sabeh et al. (2009) or Li et al. (2008), who performed *in vitro* experiments using a cross-linked native type I collagen assay to investigate the importance of MT1-MMP in cancer invasion.

Future work in developing the model will involve a parameter sensitivity analysis for the system. However, one observation that we have already noticed is that by increasing the production of TIMP2 to 10 times its original value (i.e., α*_T_* = 40) greatly reduces matrix degradation over the time interval considered and the cancer cell population increases by only 56% from its initial value (see Figure S7 in the Supplementary Material). This is due to an abundance of TIMP2 molecules binding to MT1-MMP molecules before free MT1-MMP are able to take part in activating MMP-2, as described in stage 2 of Figure 2. These observations are in line with the observed biological results of Strongin et al. (1995), Sabeh et al. (2009). We also note that for low levels of TIMP2 production, activation of MMP-2 is reduced while the concentration of active MT1-MMP is increased and the total level of ECM degradation is reduced.

We will also consider a more detailed activation system for MMP-2. While we reduced the full activation system of MMP-2 (shown in Figure 1) to the simpler system used in the model (shown in Figure 2), it is not clear if the activation system of Figure 1 captures the full range of dynamics involved in this process. Another component of the activation system which could be considered is an intermediate form of MMP-2 occurring between the proMMP-2 and fully active MMP-2. In our model, there is no intermediate form of MMP-2, since we assume proMMP-2 is in abundance and always present in the MT1-MMP:TIMP2 complex. To consider any complementary effects of further activation systems of MMP-2 on cancer cell invasion, they must first be established. Lafleur et al. (2003) have proposed that proMMP-2 binds to a different cell receptor than is modeled in our current model of the MT1-MMP:TIMP2 complex. The proMMP-2 is the processed into an intermediate form of MMP-2 before binding to the MT1-MMP:TIMP2 complex and auto-catalyzing into active MMP-2. Investigations into further activation systems by focusing on each stage of Figure 1 may be helped by the work of Nishida et al. (2008) who showed that an artificial receptor for proMMP-2 was able to take the place of the MT1-MMP:TIMP2 complex in binding proMMP-2 to the cell surface without inhibiting active MT1-MMP or MMP-2. In order to gain the full benefit of such modeling efforts (at the cell and receptor scale), we will also require to adapt the current model to take into account novel multi-scale modeling approaches, some of which have already been formulated specifically to investigate cancer invasion [cf. Trucu et al. (2013)].

## Conflict of Interest Statement

The authors declare that the research was conducted in the absence of any commercial or financial relationships that could be construed as a potential conflict of interest.

## Supplementary Material

The Supplementary Material for this article can be found online at http://www.frontiersin.org/Molecular_and_Cellular_Oncology/10.3389/fonc.2013.00070/abstract
